# Commonly Used Dose of Montmorency Tart Cherry Powder Does Not Improve Sleep or Inflammation Outcomes in Individuals with Overweight or Obesity

**DOI:** 10.3390/nu16234125

**Published:** 2024-11-28

**Authors:** Robin M. Tucker, Nahyun Kim, Eric Gurzell, Sruti Mathi, Shreya Chavva, Dharshini Senthilkumar, Olivia Bartunek, Kayla C. Fenton, Sidney J. Herndon-Fenton, Vanessa N. Cardino, Gabrielle M. Cooney, Sam Young, Jenifer I. Fenton

**Affiliations:** 1 Department of Food Science and Human Nutrition, Michigan State University, 469 S. Wilson Rd., Rm 204, East Lansing, MI 48824, USA; gurzelle@msu.edu (E.G.); chavvash@msu.edu (S.C.); senthil6@msu.edu (D.S.); bartune4@msu.edu (O.B.); fentonk2@msu.edu (K.C.F.); fentonsi@msu.edu (S.J.H.-F.); cardinov@msu.edu (V.N.C.); cooneyga@msu.edu (G.M.C.); youngs45@msu.edu (S.Y.); imigjeni@msu.edu (J.I.F.); 2 Department of Physical Education, Korea University, Seoul 02841, Republic of Korea; nahyunkim@korea.ac.kr

**Keywords:** sleep, sleep quality, inflammation, obesity, cytokine

## Abstract

Background/Objectives: Sleep problems are frequently experienced and play an important role in inflammation and disease risk. US Montmorency tart cherries (MTC) improve sleep outcomes in previous studies, but studies in individuals with overweight and obesity are lacking. Methods: A total of 34 individuals with sleep issues and overweight or obesity (BMI: 32.1 ± 7.0 kg/m^2^) were recruited to this randomized controlled, crossover study. MTC capsules (500 mg) or a placebo were taken one hour before bed for 14 days. Sleep outcomes including total sleep time, deep and REM sleep duration, nap duration, and nocturnal sleep duration were assessed using the Zmachine and/or Fitbit Inspire 3. Subjective sleep information on quality and insomnia symptoms was collected using the Pittsburgh Sleep Quality Index, the Sleep Quality Scale, and the Insomnia Severity Index. Markers of inflammation included C-reactive protein, TNF-α, and IL-6, IL-8, IL-10, and IL-17A. Results: No significant effects of MTC supplementation were observed for any of the measures of interest (*p* > 0.05 for all). Conclusions: These results suggest studies of individuals with overweight and obesity should test higher doses of MTC than those currently recommended.

## 1. Introduction

Sleep problems, including insufficient and poor-quality sleep, are widespread, costly, and are directly or indirectly linked with all 10 leading causes of death in the US [1,2,3,4,5,6,7,8], many of which are associated with inflammation [9,10,11]. Sleep plays an important role in healthy immune system function, and alterations in sleep result in inflammatory cytokine dysregulation, including interleukin-6 (IL-6), tumor necrosis factor-alpha (TNF-α), and C-reactive protein (CRP) [12,13,14]. This dysregulation can reinforce sleep issues due to circadian rhythm misalignment of these inflammatory biomarkers [12,13]. Obesity also plays a role in the development of inflammation [15,16], and individuals with obesity are more likely to report sleep problems with weight gain predictive of sleep issues [17]. Thus, sleep, inflammation, and obesity are closely connected.

The public health burdens of excess adiposity are well known, but sleep problems are also widespread. Projections suggest nearly 1 in 2 American adults will be living with obesity by 2030, and 1 in 4 will be severely obese [18]. Similarly, nearly 1 in 3 of American adults report routinely sleeping 6 h/night or less [19]. Current sleep recommendations are 7–9 h/night for adults [20]. Given that the prevalence of sleep issues is on par with that of obesity, improving sleep is an equally important public health goal.

While pharmacological approaches to addressing sleep issues exist, many individuals are interested in natural approaches to promote sleep. A survey of Canadian adults in 2009 reported that almost 20% of respondents had used an herbal or natural remedy to improve sleep while 12% used prescription medication and 8% used over the counter medication and/or alcohol [21]. Based on data from the National Health and Nutrition Examination Survey (NHANES), melatonin use increased from 0.4% in 1999–2000 to 2.1% in 2017–2018 among the US adults surveyed [22]. Given the widespread prevalence of sleep problems and consumer interest in natural alternatives to promote sleep, identification of effective interventions is warranted. One such potential intervention is the use of US Montmorency tart cherries (MTC).

A growing body of research suggests that MTC can improve sleep outcomes, including: duration [23,24], efficiency [23,24], and insomnia severity [25]. MTC likely affect sleep through an array of bioactive components known to improve both sleep and inflammation, which could serve to disrupt the sleep–inflammation–obesity cycle. These compounds include kaempferol, quercetin, melatonin, cyanidin 3-glucosylrutinoside, cyanidin 3-rutinoside, cyanydin sophoroside, and peonidin 3-glucoside [26]. MTC have demonstrated positive effects on both sleep duration [23] and quality [24], likely due, in part, to the biologically available melatonin [27,28], a sleep-promoting hormone, MTC provide [24,29]. Previously published work suggests that MTC also contain anti-inflammatory compounds that reduce dysregulation of inflammatory cytokines that interfere with sleep and/or result from sleep issues [30]. Thus, there are multiple plausible biochemical mechanisms by which MTC could disrupt the sleep–inflammation–obesity cycle.

While the limited research available suggests that MTC would have a beneficial effect on sleep and inflammatory outcomes, testing the effects of MTC on sleep and inflammation in a purposefully recruited overweight or obese population, to our knowledge, has not been conducted. One study did examine the effects of MTC juice administration on sleep in an older adult population whose average BMI was 28.1 ± 4.0 kg/m^2^ (overweight), but a population with overweight and obesity was not targeted, and only eight people completed both the intervention and control arm [23]. Sleep duration and efficiency both increased. Based on this limited evidence, the working hypothesis was that MTC would improve measures of sleep and inflammation in individuals with overweight or obesity.

## 2. Materials and Methods

**Figure 1:**
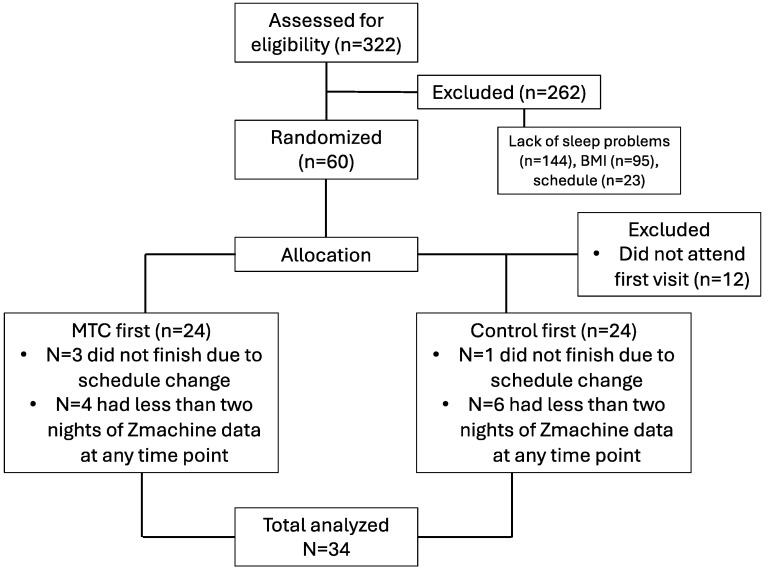
Study CONSORT diagram.

Participants were recruited through social media, flyers, and the university’s human subjects research pool. Potential participants were eligible if they were adults ages 18–50 y; had a body mass index (BMI) of ≥25.0 kg/m^2^, indicating overweight or obesity; had sleep issues as identified by a global score of ≥5 on the Pittsburgh Sleep Quality Index (PSQI) [31] and/or a score ≥ 8 on the Insomnia Severity Index (ISI) [32]; were willing to adhere to a diet low in antioxidants during the study period; agreed to wear a Fitbit Inspire 3 (Fitbit, San Francisco, CA, USA) for the duration of the study; and were willing to wear the Zmachine (General Sleep, Cleveland, OH, USA), a single-channel electroencephalograph, on selected nights (Figure 1) (see measurement information below). The study was approved by the Michigan State University Human Research Protection Program (STUDY#00008275), and all participants provided written consent. Using G*Power 3.1 (Düsseldorf, Germany), conservative assumptions of a medium effect size, alpha = 0.05, and a desired power of 80%, indicated that a total of 24 participants must complete both the intervention and control arms to test for differences between treatments.

### 2.1. Study Design

**Figure 2:**
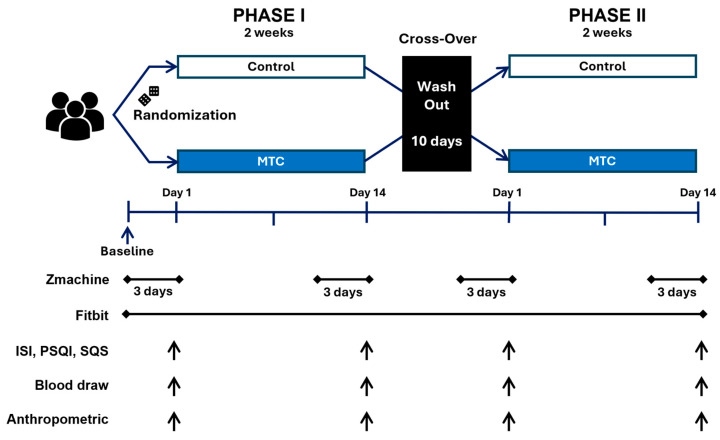
Study design. Participants were randomized to either the MTC or control arm for 14-days. Zmachine sleep data were collected for 3 consecutive days prior to the start and end of each arm. The Fitbit was worn throughout the study, but data from the same timeframe as the Zmachine were analyzed. Subjective sleep data were collected using the ISI, PSQI, and SQS at baseline and follow-up of each arm. Blood and anthropometric data were collected at baseline and follow-up of each arm. A washout period of at least 10 days was completed between each arm. Arrows indicate when data was collected.

The study was a randomized control trial with a crossover design (see Figure 2). Each arm lasted 14 days with a minimum 10-day washout period [33]. Participants came to the laboratory every Tuesday between 4:00–6:00 p.m. to minimize circadian rhythm effects [34] on the biomarkers of interest (see markers of inflammation below). This time of day has also been reported to be a period of maximal elevation of IL-6 and TNF-α in individuals with sleep problems [35]. Data collection occurred between February 2023–March 2024. This project was registered at clinicaltrials.gov (NCT05700643).

During the three days prior to the baseline lab visit for each arm, sleep data were collected using both Zmachine and Fitbit devices (see objective sleep measurements below). This was repeated three days before the final visit of each arm. Two days prior to the baseline lab visit, the low antioxidant diet was initiated and maintained during the 14-day trial period. More information about the diet has been published previously [36], but in brief, the diet restricts dietary intake of fruits and vegetables to no more than two servings per day. This dietary restriction was implemented to ensure that any changes in inflammatory biomarkers were likely attributed to the MTC supplementation rather than dietary intake [36].

### 2.2. Anthropometrics

Anthropometric information was obtained at baseline and follow-up visits. Height was measured using a stadiometer. Weight, body mass index (BMI), and percent body fat (%BF) were measured by bioelectrical impedance (TBF-400, Tanita, Tokyo, Japan).

### 2.3. Sleep Quality

Sleep quality was measured using two tools. First, the Pittsburgh Sleep Quality Index (PSQI) was used to assess study eligibility. PSQI scores can range from 0–21, and the tool reflects sleep quality over the past month. Eligible participants had a global score ≥ 5 based on a pre-screening questionnaire, as scores of 5 or greater indicate poor sleep quality [31,37]. PSQI scores were measured at all time points. The Sleep Quality Scale (SQS) was also used to measure sleep quality as it assesses sleep quality over the past week [38], rather than the past month. This timeframe was more responsive to the present study’s duration. The SQS consists of one question, “During the past 7 days, how would you rate your sleep quality overall?”, and responses are anchored with 0 (Terrible) to 10 (Excellent). SQS scores were measured at all time points.

### 2.4. Insomnia Symptoms

The Insomnia Severity Index (ISI) is a validated tool that measures perceived nocturnal and diurnal insomnia symptoms over the past two weeks [32]. Scores range from 0–28. A score ≥ 8, indicating the presence of insomnia symptoms [39], indicated eligibility. ISI scores were measured at all time points.

### 2.5. Objective Sleep Measures

Two tools were used to objectively measure sleep outcomes. The Zmachine (General Sleep, Cleveland, OH, USA) is a single-channel research-grade electroencephalograph that participants wear at home. The Zmachine provides information about time spent in sleep stages (deep and REM) and time to fall asleep, and has been shown to substantially agree with polysomnography (PSG), the gold standard for sleep measurements [40,41,42]. The Zmachine was worn for three nights at baseline and follow-up for each arm. Total sleep time (TST) as well as time spent in deep (stage N3) and REM sleep were collected. These stages are considered to be the more restorative stages of sleep [43].

Because the Zmachine requires the participant to remember to use it, we also provided participants with a Fitbit Inspire 3 (Fitbit Inc., San Francisco, CA, USA) to track sleep. Fitbits have been shown to substantially agree with PSG, as well as research-grade actigraphy [40,42]. Further, a recent meta-analysis concluded that Fitbits are an acceptable alternative to PSG when measuring total sleep duration [44]. Total sleep time (TST) was measured using the Fitbit Inspire 3 at the same time points as the Zmachine. We compared the agreement between the data derived from the Zmachine and Fitbit Inspire 3 to confirm the participants were wearing the Zmachine as instructed. The Fitbit also collected napping data. Fitbit data were broken down by nocturnal sleep, naps, and total sleep time (nocturnal sleep plus naps) to provide a more complete understanding of sleeping habits.

### 2.6. Markers of Inflammation

Due to their associations with sleep issues and obesity, the following inflammatory biomarkers were selected for measurement: C-reactive protein (CRP) [45,46], Interleukin-6 (IL-6) [35,45,47,48,49], Interleukin-8 (IL-8) [47], Interleukin-10 (IL-10), Interleukin-17A (IL-17A) [48], and tumor necrosis factor-alpha (TNF-α) [35,47,49,50]. Serum was stored at −80 °C within 30 min of acquisition. Serum biomarkers were measured using enzyme-linked immunosorbent assay (ELISA) Duoset^®^ kits from R&D Systems (Minneapolis, MN, USA) with no modification to the manufacturer protocol. Colorimetric optical density (O.D.) data were collected using an Agilent Biotek Synergy HT Plate Reader (Winooski, VT, USA).

### 2.7. Intervention

Based on the appropriate arm, participants were instructed to consume a 500 mg dose of 100% MTC powder (tart cherry powder, Shoreline Fruit, Williamsburg, MI, USA) or 460 mg corn starch placebo in the form of two pills [51] one hour [52] before their desired bedtime. The MTC powder consisted of whole US Montmorency tart cherries without additives or excipients. The 500 mg dose of MTC is similar in terms of anthocyanin and polyphenol content to 2 ounces of MTC concentrate, [51] an amount shown to improve sleep outcomes [23,24], but the powder retains melatonin that can be lost with concentrate processing [26]. MTC powder was selected instead of MTC juice or concentrate for several reasons: MTC concentrate has been shown to contain lower amounts of melatonin than MTC powder [26]; products processed with sugar demonstrate reduced amounts of anthocyanin and phenolic compounds [26]; and juice vehicles could cause potential issues with sleep fragmentation due to nocturnal urination.

### 2.8. Statistical Analysis

Statistical analysis of the data was performed with JAMOVI version 2.4.14 (Sydney, Australia). The normality of residuals was assessed using Q-Q plots and the Shapiro–Wilk test, while their variance was evaluated through scatter plots. All sleep data residuals satisfied the assumption of normality and, thus, were analyzed using a linear mixed model. Linear mixed models were employed to explore the primary impacts of time, treatment, and their interaction (2 time × 2 treatment). We also employed a linear mixed model with treatment, time, and their interaction as fixed effects to evaluate the primary effects and interactions of treatments over time. A random intercept was included to account for individual variability, capturing baseline differences and accommodating the repeated measures design for accurately assessing treatment effects against individual baseline characteristics in crossover studies. Upon detecting a significant F ratio, we proceeded with post hoc comparisons using the Bonferroni correction method.

Since the inflammatory markers (TNF-α, IL-6, IL-8, IL-10, IL-17A, CRP) did not follow a normal distribution, we utilized natural log-transformed values for all statistical analyses. Inflammation data were analyzed using generalized linear models (GLMs). GLMs were utilized to investigate the main effects of time, treatment, and their interaction (2 time × 2 treatment).

Statistical significance for all hypothesis tests was determined using a threshold of *p* < 0.05, with tests being two-tailed to allow for the detection of effects in either direction. Data are represented as mean ± standard deviation (SD).

## 3. Results

The baseline characteristics of the study population are presented in Table 1. A total of 34 participants completed the study; 16 individuals (5 male, 11 female) completed the MTC arm first, and 18 individuals completed the placebo arm first (4 male, 14 female). No differences in age (32.9 ± 11.4 y vs. 32.3 ± 10.5), BMI (34.5 ± 8.9 kg/m^2^ vs. 30.0 ± 3.8 kg/m^2^), or %BF (38.8 ± 10.5% vs. 36.9 ± 7.5%) were observed at baseline (*p* > 0.05 for all). There were no significant differences in sleep duration (TST, deep sleep, REM sleep) or inflammation biomarkers (TNF-α, IL-17A, IL-6, IL-8, IL-10, CRP) across the different groups at baseline (*p* > 0.05 for all). There was no change in %BF or BMI throughout the experimental period across all treatments and times (*p* > 0.05).

### 3.1. Sleep Outcomes

As shown in Table 2, there were no significant differences in objective sleep outcomes measured using the Zmachine (TST, deep sleep, REM sleep) between groups, over time, or in the interaction between group and time (*p* > 0.05 for all). Additionally, there were no statistically significant differences observed in nocturnal sleep duration, nap duration, or total sleep time measured by the Fitbit across groups and time periods. The intake of MTC for 14 days did not result in any significant changes in objective sleep outcomes.

In terms of subjective sleep measures, MTC intake decreased ISI scores compared to baseline (t = −2.98, *p* < 0.05), but there was no significant difference between treatment groups (t = 0.11, *p* = 0.92). Similarly, the PSQI score also decreased after intake of MTC (t = 0.51, *p* < 0.001), but there was no difference between treatment groups (t = 0.66, *p* = 0.51). SQS showed no significant differences in time, group, or interaction (Table 3).

**Figure 3:**
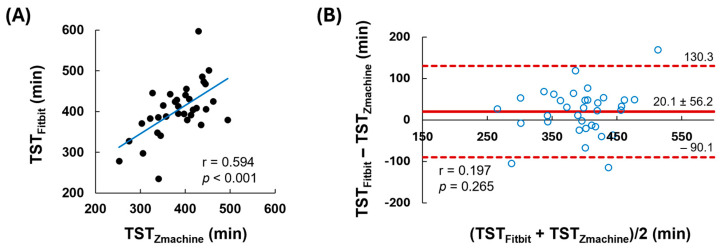
Correlation and Bland-Altman plot of TST between Fitbit and Zmachine. Panel (**A**) is the correlation plot for the TST obtained from Fitbit and Zmachine. Panel (**B**) shows the Bland-Altman plot, which assesses the agreement between the two devices. The solid red line represents the mean difference (bias), and the dashed red lines indicate the limits of agreement (mean ± 2SD).

To ensure the reliability and accuracy of TST, we compared the TST measured at baseline using both the Fitbit and Zmachine. The correlation (Figure 3A) shows a significant relationship between the TST measurements from Fitbit and Zmachine (r = 0.594, *p* < 0.001). The Bland–Altman plot evaluates the agreement of TST measurements between the Fitbit and Zmachine (Figure 3B). The limits of agreement (LoA) between the two methods (mean difference ± 2SD) were calculated using the Bland–Altman method, which allowed for the determination of the average difference between pairs of variables, representing the overall bias. The majority of the data lie within the LoA, and the mean bias between TST_Fitbit_ and TST_Zmachine_ was 20.1 min. Furthermore, the correlation between this bias (Fitbit − Zmachine) and the average (Fitbit + Zmachine/2) was not significant, which indicates there is no proportional bias in the values between the two measurements.

Table 4 shows comparison and agreement between Fitbit and Zmachine data for TST. To assess the reliability and agreement of TST measurements between the Fitbit and Zmachine, intraclass correlation coefficients (ICC) and 95% confidence intervals (95% CI) were utilized. The reliability of the ICC was determined according to Landis’s criterion [53]. ICC was found to be 0.738, which indicates a substantial level of agreement and suggests the Fitbit and Zmachine measurements have a high degree of accuracy. The trend in bias is expressed by the linear regression line for the data of Bland-Altman plots, which is calculated as intercept + slope multiplied by the mean of Fitbit and Zmachine (M). The *p*-value for the trend in bias was not significant (*p* = 0.265), indicating the absence of systematic bias between the two methods.

### 3.2. Inflammation Biomarkers

None of the inflammatory markers (TNF-α, IL-6, IL-8, IL-10, IL-17A, or CRP) showed significant differences between pre- and post-tart cherry intake. There were no differences observed between the control and tart cherry-treated groups, nor was there any interaction between time and treatment (*p* > 0.05 for all) (Table 5).

## 4. Discussion

This study examined the effects of US Montmorency tart cherry powder on sleep outcomes. Despite providing a dose shown to improve sleep outcomes in other studies [23,24,25], there were no appreciable effects observed. It is possible that MTC powder is less effective in the targeted population, i.e., individuals presenting with overweight or obesity, and that a higher dose is needed.

Other studies indicate that MTC improve sleep outcomes [23,24,25], likely through multiple mechanisms. The best studied of these mechanisms are increased melatonin availability and cytokine modulation. First, MTC contain phytomelatonin [29], which refers to melatonin derived from plant origins rather than animal origins or chemical synthesis [54]. Regardless of its origin, melatonin is an endogenously produced hormone that reduces alertness, which promotes sleep [55]. Melatonin can also effectively promote sleep when taken as a supplement or when foods rich in melatonin are consumed [24,56]. Melatonin levels peak approximately one hour after oral administration [52]. While melatonin doses provided by MTC are lower than those considered physiologically effective, it is posited that the melatonin, working synergistically with the additional anti-inflammatory compounds present, discussed next, is what leads to the positive outcomes reported [24,30].

A second putative sleep-promoting pathway is via anti-inflammatory compounds in MTC that can modulate sleep-promoting cytokines. For example, insufficient sleep increases serum concentrations of CRP, TNF-α, and IL-6, which are sleep promoting cytokines, but it does so out of phase; these compounds are increased in the afternoon, promoting daytime sleepiness, rather than at night [57,58,59,60]. Further, IL-6 is associated with sleep architecture and depth of sleep; lower levels of nocturnal IL-6 result in less time spent in the more restorative stages of sleep [59,60]. Poorer sleep quality has been associated with higher levels of daytime IL-6, IL-8, and TNF-α [47] while sleep restriction has been linked with higher levels of CRP and IL-17 [48]. MTC contain a variety of potent anti-inflammatory compounds, including kaempferol, quercetin, melatonin, among others [26], that have been shown to be effective in increasing total antioxidant capacity and reducing inflammation [61,62]; however, one recent report suggested melatonin did not reduce inflammation in a cell model [63]. Baseline mean TNF-α concentrations of 1.8 ± 8.5 pg/mL in the study population were similar to median concentrations observed in subjects without (1.6 (1.2–2.1 pg/mL)) and with obesity (1.8 (1.4–2.2 pg/mL)) in a study of cytokine profiles by BMI in individuals who were matched for age and gender [64]. IL-6 concentrations observed in the study population (3.0 ± 10.1 pg/mL (range 0–53.9 pg/mL)) were also similar to a pooled estimate of IL-6 of 5.186 pg/mL ([CI]: 4.631, 5.740) and range (0–43.5 pg/mL) in a meta-analysis of healthy populations [64]. IL-10 concentrations were 5.0 ± 15.8 pg/mL in the present population, with other reports showing IL-10 ranging from 1.3 pg/mL to greater than 74.4 pg/mL [65]. Previous reports on IL-10 are heterogeneous, with reduced levels in subjects with obesity [66], elevated levels in females with obesity [67], or elevations associated with visceral fat loss [65]. In general, the levels of IL-8 (0.7 ± 2.3 pg/mL) and IL-17A (0.3 ± 1.2 pg/mL) in this population were lower than expected [68,69]. For example, one study reported mean IL-8 concentrations of 4.3 ± 1.4 pg/mL in a population with obesity [69], and another described mean IL-17A levels in healthy subjects (10.1 ± 3.0 pg/mL) and patients with severe obstructive sleep apnea–hypopnea syndrome (20.3 ± 3.9 pg/mL) as higher than those observed in the present study [70]. The reason for these discrepancies is unknown. The levels of cytokines at baseline in this population were generally consistent with reported values, and the ELISA assays used were sufficient in sensitivity for the expected values. CRP levels increased with obesity as expected [71]. The study population had an average BMI of 32.1 kg/m^2^ at baseline and a corresponding mean CRP level of 4.4 ± 10.0 mg/L, consistent with an adjusted geometric mean CRP level of 3.22 mg/L in a study of inflammatory markers and BMI [71]. Despite having a study population with cytokine levels relatively consistent with similar populations, MTC supplementation did not alter or improve inflammatory biomarker profiles.

Obesity and sleep problems are both associated with undesirable inflammation profiles. Expanding adipose tissue in obesity drives a variety of metabolic changes, including elevated cytokine production [72]. Sleep problems result in cytokine dysregulation, as just discussed [60,73], even in lean individuals [12,35,48,49]. While sleep issues can quickly alter these biomarkers, with cytokine dysregulation observed in a week or less [12,49], weight loss is required to reduce obesity-triggered inflammation [15]. Our participants did not experience weight loss or a change in body composition during the course of the study.

As stated above, multiple studies indicate that MTC improved sleep outcomes [23,24,25]. These studies are included in a recent meta-analysis that concluded tart cherries improve objectively measured total sleep time and sleep efficiency, but of the eight included studies, three used cherries other than MTC and one used a dietary supplement containing other ingredients besides tart cherries [74,75,76,77]. Of these four studies, three reported sleep improvements [75,76,77]. Thus, more work is needed to conclusively determine the effectiveness of MTC on sleep outcomes in various populations.

Despite some studies indicating positive effects, our findings of no effect of MTC on sleep outcomes agree with other reports. Neither MTC juice nor supplementation improved sleep duration or quality more than placebo in one recent study [51]. That study used the same MTC powder dose as the current study and supplemented for 30 days. Others reported no effect of MTC supplementation after 4 weeks on inflammatory markers, but the study samples consisted of healthy individuals with low levels of inflammation at baseline [51,78]; thus, there was limited room for improvement. Another study indicated no effect of MTC juice administration on sleep, but juice rather than MTC powder was used [23]. Since MTC concentrate has been shown to contain lower amounts of melatonin than MTC powder [26] and may have reduced amounts of anthocyanin and phenolic compounds [26], these factors could have reduced the effectiveness of the intervention [26]. Another study exploring the effects of concentrate consumption for three months observed no improvements in sleep quality [79]. Given the totality of the findings on MTC and sleep, the results are mixed, and it is worth further exploration to better understand the reasons for these discrepancies in order to optimize MTC use for sleep.

We expected that MTC supplementation would improve sleep outcomes, including sleep quality, sleep duration, and insomnia symptoms, while also improving markers of inflammation. None of these hypothesized effects were observed. Individuals with overweight or obesity may require higher doses to observe effects. It is also possible that a longer duration of supplementation is necessary to achieve positive results. However, a different study supplemented for 30 days but also observed no effects on inflammation [51]. More research on optimization of timing and dosage is needed.

As with any study, there are strengths and limitations. The RCT design with crossover is a strength. Further, the study utilized a variety of both subjective and objective measures of sleep, selected to encompass a holistic representation of sleep problems. These measures are among the most comprehensive measures used in the studies that have previously been completed in this research area. Limitations include a lack of confirmation that participants actually consumed the pills provided.

## 5. Conclusions

The effects of MTC supplementation among individuals with overweight or obesity were tested in this study. Analyses demonstrated that MTC supplementation had no effect on sleep or inflammation markers in this study. The observation of no effect despite using a previously validated dose suggests individuals with higher BMI may need higher levels of supplementation to achieve the effects reported in other studies.

## Figures and Tables

**Table 1:** Baseline characteristics of study population.

Characteristics	Total N = 34 (9 M, 25 F)	1st MTC, 2nd Placebo N = 16 (5 M, 11 F)	1st Placebo, 2nd MTC N = 18 (4 M, 14 F)	*p* Value
Age (y)	32.6 ± 10.7	32.9 ± 11.4	32.3 ± 10.5	0.735
Weight (kg)	91.7 ± 23.5	98.8 ± 29.5	85.4 ± 14.6	0.192
Height (cm)	168.3 ± 10.4	168.2 ± 10.8	168.4 ± 10.4	0.748
BMI (kg/m^2^)	32.1 ± 7.0	34.5 ± 8.9	30.0 ± 3.8	0.120
% Body fat	37.8 ± 9.0	38.8 ± 10.5	36.9 ± 7.5	0.799
TST (min/day)	383.8 ± 56.5	369.8 ± 65.0	396.3 ± 46.2	0.545
Deep sleep (min/day)	86.5 ± 25.3	81.3 ± 81.3	91.1 ± 23.8	0.381
REM sleep (min/day)	96.2 ± 29.7	95.1 ± 35.5	97.2 ± 24.5	0.795
TNF-α (pg/mL)	1.8 ± 8.5	3.1 ± 12.1	0.7 ± 2.1	0.506
IL-6 (pg/mL)	3.0 ± 10.1	4.9 ± 13.9	1.2 ± 4.2	0.409
IL-8 (pg/mL)	0.7 ± 2.3	0.4 ± 1.1	0.9 ± 3.0	0.160
IL-10 (pg/mL)	5.0 ± 15.8	9.0 ± 22.3	1.5 ± 4.0	0.246
IL-17A (pg/mL)	0.3 ± 1.2	0.1 ± 0.4	0.6 ± 1.7	0.235
CRP (mg/L)	4.4 ± 10.0	4.8 ± 6.7	4.1 ± 12.5	0.976

Data are presented as mean ± standard deviation. MTC: Montmorency tart cherry; BMI: body mass index; TST: total sleep time; REM: rapid eye movement.

**Table 2:** Within- and between-group effects of control and tart cherry group on sleep outcomes.

Variables	Time	Treatment			Estimate	SE	95% CI		*df*	t	*p*
		Control	MTC				Lower	Upper			
Zmachine											
TST	Pre	375.4 ± 59.2	366.8 ± 60.7	Time	−10.11	7.43	−24.80	4.58	66.0	−1.36	0.18
	Post	366.2 ± 59.4	355.8 ± 59.0	treatment	−9.52	12.25	−33.80	14.72	66.0	−0.78	0.44
				Time × treatment	−1.75	14.85	−31.10	27.63	66.0	−0.12	0.91
Deep sleep	Pre	82.1 ± 27.6	78.5 ± 26.4	Time	−1.38	2.79	−6.91	4.15	66.0	−0.49	0.62
	Post	77.7 ± 27.7	80.2 ± 29.7	treatment	−0.57	6.16	−12.76	11.62	66.0	−0.09	0.93
				Time × treatment	6.18	5.59	−4.87	17.23	66.0	1.11	0.27
REM sleep	Pre	95.9 ± 28.2	89.88 ± 36.8	Time	0.43	0.80	−1.16	2.02	66.0	0.54	0.59
	Post	98.0 ± 41.2	87.13 ± 32.5	treatment	0.51	1.68	−2.81	3.82	66.0	0.30	0.76
				Time × treatment	2.12	1.61	−1.06	5.29	66.0	1.32	0.19
Fitbit											
Nocturnal	Pre	391.0 ± 98.4	385.1 ± 89.9	Time	4.56	23.2	−41.3	50.4	60.2	0.20	0.85
	Post	394.9 ± 98.7	390.3 ± 56.0	treatment	−31.64	42.1	−114.9	51.6	61.8	−0.75	0.46
				Time × treatment	−40.81	46.3	−132.5	50.9	60.2	−0.88	0.38
Nap	Pre	6.5 ± 14.4	12.1 ± 23.0	Time	3.68	10.95	−18	25.34	65.4	0.34	0.74
	Post	13.3 ± 25.4	8.0 ± 21.7	treatment	0.07	11.62	−22.9	23.06	65.6	0.01	0.99
				Time × treatment	−34.47	21.89	−77.8	8.85	65.4	−1.57	0.12
Total Sleep	Pre	397.6 ± 98.5	397.2 ± 92.7	Time	7.37	23.3	−38.8	53.6	59.7	0.32	0.75
	Post	408.2 ± 99.4	398.2 ± 51.4	treatment	−30.65	41.0	−111.7	50.4	61.2	−0.75	0.46
				Time × treatment	−76.37	46.7	−168.7	16	59.7	−1.63	0.11

Data are presented as mean ± standard deviation. MTC: Montmorency tart cherry; TST: total sleep time; REM: rapid eye movement; SE: standard error; CI: confidence interval.

**Table 3:** Within- and between-group effects of control and tart cherry group on sleep quality.

Variables	Time	Treatment			Estimate	SE	95% CI		*df*	t	*p*
		Control	MTC				Lower	Upper			
Survey											
ISI	Pre	11.85 ± 5.27	12.21 ± 5.39	Time	−1.23	0.41	−2.05	−0.41	64.3	−2.98	<0.05 *
	Post	10.84 ± 4.62	10.82 ± 5.06	treatment	0.13	1.18	−2.20	2.45	65.1	0.11	0.92
				Time × treatment	−0.46	0.83	−2.10	1.18	64.3	−0.56	0.58
PSQI	Pre	8.58 ± 3.35	8.88 ± 3.25	Time	−1.09	0.31	−1.69	−0.48	63.9	−3.55	<0.001 *
	Post	7.33 ± 3.05	8.03 ± 2.79	treatment	0.46	0.70	−0.92	1.85	65.3	0.66	0.51
				Time × treatment	0.31	0.61	−0.90	1.52	63.9	0.51	0.61
SQS	Pre	4.94 ± 1.58	5.21 ± 1.63	Time	0.15	0.19	−0.23	0.53	62.0	0.78	0.44
	Post	5.12 ± 1.93	5.21 ± 1.65	treatment	0.14	0.38	−0.61	0.88	65.9	0.36	0.72
				Time × treatment	−0.10	0.39	−0.86	0.67	62.0	−0.25	0.81

Data are presented as mean ± standard deviation. MTC: Montmorency tart cherry; ISI: Insomnia Severity Index; PSQI: Pittsburgh Sleep Quality Index; SQS: Sleep Quality Scale; SE: standard error; CI: confidence interval. * Indicates significance.

**Table 4:** Comparison and agreement between Fitbit and Zmachine on total sleep time (TST).

	TST (Mean ± SD)	Difference (95% CI)	Trend in Bias [*p* Value]	Agreement (ICC, 95% CI)
Zmachine	383.8 ± 56.5			
Fitbit	403.9 ± 66.5	20.1 (0.525–39.7)	−59.6 + 0.2 M [0.265]	0.738 (0.475–0.869)

TST: total sleep time; CI: confidence interval; ICC: intra-class correlation coefficient. Trend in bias: The linear regression line for the data of Bland-Altman plots is expressed as intercept + slope × mean of Fitbit and Zmachine (M).

**Table 5:** Within- and between-group effects of control and tart cherry group inflammation biomarkers.

Variables	Time	Treatment			Estimate	SE	Odds Ratio	Odds Ratio 95% CI		z	*p*
		Control	MTC					Lower	Upper		
Inflammation biomarkers											
TNF-ɑ (pg/mL)	Pre	1.80 ± 7.40	2.01 ± 8.45	Time	−0.01	0.06	0.99	0.88	1.12	−0.16	0.87
	Post	1.85 ± 8.47	2.53 ± 9.02	treatment	0.03	0.06	1.03	0.91	1.16	0.41	0.68
				Time × treatment	0.03	0.12	1.03	0.81	1.31	0.22	0.83
IL-6 (pg/mL)	Pre	2.92 ± 8.57	3.24 ± 10.29	Time	−0.02	0.07	0.98	0.85	1.14	−0.26	0.80
	Post	3.05 ± 9.87	2.58 ± 7.45	treatment	0.00	0.07	1.00	0.86	1.15	−0.05	0.96
				Time × treatment	0.02	0.15	1.02	0.76	1.37	0.16	0.87
IL-8 (pg/mL)	Pre	0.78 ± 2.35	0.67 ± 1.58	Time	0.00	0.04	1.00	0.85	1.14	−0.26	0.80
	Post	0.42 ± 1.18	0.96 ± 1.97	treatment	0.03	0.04	1.03	0.86	1.15	−0.05	0.96
				Time × treatment	0.07	0.08	1.08	0.76	1.37	0.16	0.87
IL-10 (pg/mL)	Pre	5.08 ± 16.92	4.49 ± 15.65	Time	0.00	0.09	1.00	0.85	1.19	0.04	0.97
	Post	4.94 ± 15.48	3.54 ± 9.98	treatment	−0.04	0.09	0.96	0.81	1.14	−0.45	0.65
				Time × treatment	0.07	0.17	1.07	0.76	1.50	0.38	0.70
IL-17A (pg/mL)	Pre	0.38 ± 1.24	0.07 ± 0.28	Time	0.01	0.02	1.01	0.97	1.05	0.32	0.75
	Post	0.36 ± 0.90	0.15 ± 0.38	treatment	−0.03	0.02	0.97	0.93	1.01	−1.59	0.11
				Time × treatment	0.00	0.04	1.00	0.92	1.08	0.01	1.00
CRP (mg/L)	Pre	4.29 ± 9.90	3.18 ± 5.24	Time	−0.02	0.07	0.98	0.85	1.13	−0.25	0.80
	Post	3.23 ± 6.00	3.12 ± 5.68	treatment	−0.01	0.07	0.99	0.86	1.14	−0.13	0.90
				Time × treatment	−0.02	0.15	0.98	0.73	1.31	−0.15	0.88

Data are presented as mean ± standard deviation. MTC: Montmorency tart cherry; SE: standard error; CI: confidence interval.

## Data Availability

The raw data supporting the conclusions of this article will be made available by the authors on request.
